# The Arctic in the Twenty-First Century: Changing Biogeochemical Linkages across a Paraglacial Landscape of Greenland

**DOI:** 10.1093/biosci/biw158

**Published:** 2017-02-01

**Authors:** N. John Anderson, Jasmine E. Saros, Joanna E. Bullard, Sean M. P. Cahoon, Suzanne McGowan, Elizabeth A. Bagshaw, Christopher D. Barry, Richard Bindler, Benjamin T. Burpee, Jonathan L. Carrivick, Rachel A. Fowler, Anthony D. Fox, Sherilyn C. Fritz, Madeleine E. Giles, Ladislav Hamerlik, Thomas Ingeman-Nielsen, Antonia C. Law, Sebastian H. Mernild, Robert M. Northington, Christopher L. Osburn, Sergi Pla-Rabès, Eric Post, Jon Telling, David A. Stroud, Erika J. Whiteford, Marian L. Yallop, Jacob C. Yde

**Affiliations:** N. John Anderson (n.j.anderson@lboro.ac.uk) is affiliated with the Department of Geography at Loughborough University in Loughborough, UK. Jasmine E. Saros, is affiliated with the School of Biology & Ecology at the University of Maine in Orono, Maine. Joanna E. Bullard, is affiliated with the Department of Geography at Loughborough University in Loughborough, UK. Sean M.P. Cahoon, was at the Department of Biology at Penn State University, in University Park, Pennsylvania. He is presently affiliated with the Environment and Natural Resources Institute at the University of Alaska Anchorage, AK. Suzanne McGowan is affiliated with the School of Geography at the University of Nottingham in Nottingham, UK. Elizabeth A. Bagshaw is affiliated with the Earth and Ocean Sciences at Cardiff University in Cardiff, UK. Christopher D. Barry, is affiliated with the School of Biological Sciences at Queen's University in Belfast, UK. Richard Bindler is affiliated with the Department of Ecology and Environmental Science at Umeå University in Umeå, Sweden. Benjamin T. Burpee is affiliated with the School of Biology & Ecology at the University of Maine in Orono, Maine. Jonathan L. Carrivick, is affiliated with the School of Geography at the University of Leeds in Leeds, UK. Rachel A. Fowler, is affiliated with the School of Biology & Ecology at the University of Maine in Orono, Maine. Anthony D. Fox is affiliated with the Department of Bioscience, at Aarhus University in Rønde, Denmark. Sherilyn C. Fritz is affiliated with the Department of Earth and Atmospheric Sciences at the University of Nebraska in Lincoln, Nebraska. Madeleine E. Giles, is affiliated with the School of Biological Sciences at the University of Essex in Colchester, UK. Ladislav Hamerlik, was affiliated with the Department of Biology and Ecology at Matthias Belius University in Banska Bystrica, Slovakia. He is presently affiliated with the Institute of Geological Sciences, Polish Academy of Sciences, Warsaw, Poland Thomas Ingeman-Nielsen is affiliated with the Department of Civil Engineering at the Technical University of Denmark in Kongens Lyngby, Denmark. Antonia C. Law is affiliated with the Department of Geography, Geology and the Environment at Keele University in Keele, UK. Sebastian H. Mernild is affiliated with the Nansen Environmental and Remote Sensing Center, Bergen, Norway. He also has positions at Faculty of Engineering and Science, Sogn og Fjordane University College, Sogndal, Norway and Antarctic and Sub-Antarctic Program, Universidad de Magallanes, Punta Arenas, Chile. Faculty of Engineering and Science at Sogn og Fjordane University College in Sogndal, Norway. Robert M. Northington is affiliated with the School of Biology & Ecology at the University of Maine in Orono, Maine. Christopher L. Osburn is affiliated with the School of Marine, Earth, and Atmospheric Sciences at NC State University, Raleigh, North Carolina. Sergi Pla-Rabès is affiliated with the Centre de Recerca Ecològica i Aplications Forestals in Cerdanyola del Vallés, Spain. Eric Post is affiliated with the Department of Wildlife, Fish, & Conservation Biology at the University of California in Davis, California. Jon Telling was affiliated with the School of Geographical Sciences at the University of Bristol in Bristol, UK. He is presently affiliated with the School of Civil Engineering and Geosciences, Newcastle University, UK. David A. Stroud is affiliated with the UK Joint Nature Conservation Committee in Peterborough, UK. Erika J. Whiteford is affiliated with the Department of Geography at Loughborough University in Loughborough, UK. Marian L. Yallop is affiliated with the School of Biological Science, at University of Bristol in Bristol, UK. Jacob C. Yde is affiliated with the Faculty of Engineering and Science at Sogn og Fjordane University College in Sogndal, Norway.

**Keywords:** tundra, lake, carbon, permafrost, aeolian

## Abstract

The Kangerlussuaq area of southwest Greenland encompasses diverse ecological, geomorphic, and climate gradients that function over a range of spatial and temporal scales. Ecosystems range from the microbial communities on the ice sheet and moisture-stressed terrestrial vegetation (and their associated herbivores) to freshwater and oligosaline lakes. These ecosystems are linked by a dynamic glacio-fluvial-aeolian geomorphic system that transports water, geological material, organic carbon and nutrients from the glacier surface to adjacent terrestrial and aquatic systems. This paraglacial system is now subject to substantial change because of rapid regional warming since 2000. Here, we describe changes in the eco- and geomorphic systems at a range of timescales and explore rapid future change in the links that integrate these systems. We highlight the importance of cross-system subsidies at the landscape scale and, importantly, how these might change in the near future as the Arctic is expected to continue to warm.

**Arctic ecosystems have undergone major changes** over the past century (Hinzman et al., 2013). Much of this change is driven by higher temperatures, a warming that is enhanced relative to lower latitudes but other stressors, notably atmospheric deposition of reactive nitrogen (N) and other pollutants, also have profound ecological effects (Post et al., 2009, Wookey et al., 2009). Much of the focus in Arctic geomorphic and ecological research has been on the response of individual units (e.g., floodplain, population, or community) to climate and atmospheric changes. Many of the ecological responses have been relatively predictable in terms of our understanding of the underlying processes—that is, altered phenology, longer growing seasons, the greening (i.e., increased plant biomass) of terrestrial ecosystems, range expansion or contraction of plants and animals, and altered soil microbial activity associated with deepening active layers (Sturm et al., 2001; Schuur et al., 2008). Ecosystem feedbacks and their impact on the climate system are important (Wookey et al., 2009), largely because of the large carbon (C) stores (McGuire et al., 2009), but the focus is often only within individual or limited components of the Arctic system (e.g., vegetation–soils or soils–permafrost; Wookey et al., 2009).

In the broader ecological literature, the importance of climate-driven alterations to connections across ecosystems is increasingly recognized (Greig et al., 2012). Resources such as carbon, nutrients, and water are exchanged across ecosystems, and the exchange of these “subsidies” can be tightly coupled (Nakano and Murakami 2001), raising the need to better understand the pools and their linkages across the landscape. Wookey and colleagues (2009) highlighted the impact of climate and N deposition on altered terrestrial ecosystem processes and community structure and therefore the broader climate system. Overpeck and colleagues (2005) indicated the importance of feedbacks between key “hubs” in the Arctic system and possible changes as key components (e.g., sea ice or permafrost) are lost. Although the Arctic is increasingly seen as an integrated system (Hinzman et al., 2013), much of the focus on cross-system connections has been on the one-way delivery of water, carbon, and nutrients from glaciers to rivers to marine systems (Hawkings et al., 2015; O'Neel et al., 2015). The linkages across continental systems (glacier, terrestrial, and freshwater), as well as the myriad feedbacks among them, are much less clear, despite their likely importance in shaping how the landscape responds to climate and atmospheric deposition; the physical transitions between ice-sheet, terrestrial, and marine ecosystems are often unambiguous on Greenland.

In the Arctic, glaciated landscapes provide model systems in which to explore the complexity of cross-system physical and biogeochemical linkages (Anderson SP 2007). The past and present influences of glaciers impart signature effects on northern landscapes, layered on top of typical Arctic features (e.g., permafrost) that have major implications for weathering rates, geochemical cycling, and organic C sequestration (Anderson SP 2007). Ice sheets and glaciers are also recognized as distinct ecosystems (Anesio and Laybourn-Parry 2012) and microbial-dominated biogeochemistry can affect adjacent terrestrial and aquatic ecosytems (Hodson et al., 2008). Active glaciers produce rock flour and provide an important biogeochemical interface with the atmosphere, concentrating and storing water, carbon, nutrients and pollutants. These materials are released into the proglacial floodplain, a key feature of the associated paraglacial landscape (defined as a landscape that is directly conditioned by former glaciation and deglaciation). These floodplains are major components of the geomorphic system that couple glaciers and ice sheets to wider sedimentary environments (Bullard 2013). They provide sediments that are transported by fluvial and aeolian processes within the landscape at a range of timescales. In turn, the aeolian dispersal of dust from these floodplains delivers nutrient subsidies to terrestrial and freshwater systems as well as back to the glacier itself. These systems provide complex but rich opportunities in which to assess physical and biogeochemical linkages and feedbacks across Arctic landscapes and to examine how these may change in the future.

The Arctic has witnessed some of the most rapid, nonlinear environmental change in the last 20 years, with perhaps the best example of this being Greenland, where mean annual air temperatures between 2007 and 2012 were 3°C higher than averages from 1979 to 2000 (Mayewski et al., 2014). Greenland Ice Sheet (GrIS) mass losses are more than 100% higher after 1996 compared with the period between 1958 and 1996 (van den Broeke et al., 2009), with an extreme melt event on the GrIS in 2012 (e.g., Hanna et al., 2014). During the last decade, the North Atlantic and Arctic oceans have experienced the most drastic loss of sea ice ever recorded (Parkinson and Comiso 2013), coinciding with regional increases in marine net primary production (Arrigo et al., 2008). However, sea-ice loss is also changing stratification patterns, which influences the vertical transfer of nutrients and therefore phytoplankton production (Ardyna et al., 2014). Similar changes to lake stratification are also occurring with associated implications for community structure. Sulfur deposition products originating from marine phytoplankton have increased with these changes in sea ice (Sharma et al., 2012). Collectively, it is quite apparent that in the twenty-first century, we have entered a period of rapid environmental change in the Arctic.

Here we synthesize research from the area around Kangerlussuaq, southwest Greenland, at a range of temporal and spatial scales in a cross-system, multidisciplinary approach. The area of focus spans from the margin of the GrIS to the associated paraglacial landscape, situated in the largest ice-free margin of the country (figure 1). Our goal is to begin to delineate the resource pools and cross-system connections in this region to enable greater understanding of future climate-driven changes to this region.

**Figure 1.**
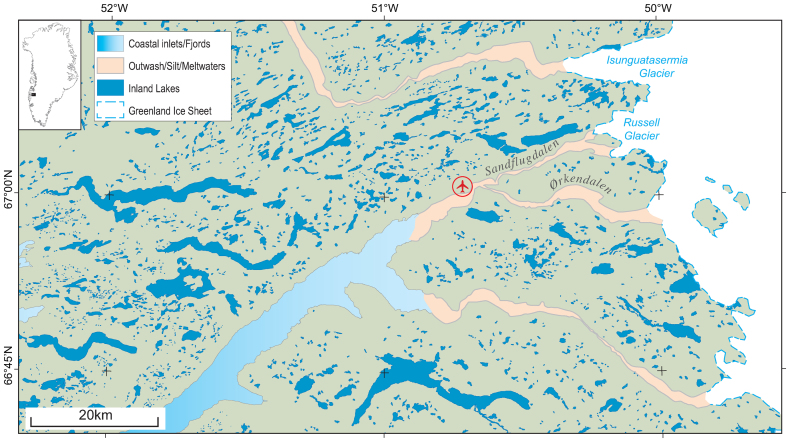
The area around Kangerlussuaq airport, showing the high density of lakes and their juxtaposition to the Greenland ice sheet, its outlet glaciers, and the outwash plains. Abbreviations: km, kilometers; N, north; W, west; degrees.

## Kangerlussuaq: The Arctic and global change encapsulated

**Figure 2.**
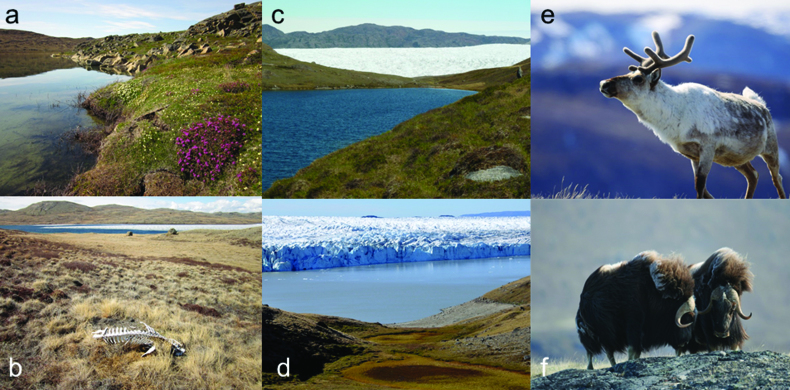
Key components of the Kangerlussuaq ecosystems. (a) Dwarf shrub tundra (photograph: John Anderson). (b) Steppe adjacent to the GrIS (photograph: John Anderson). (c) Rapid ecotonal transitions adjacent to the Isunguata Sermia glacier (see figure 1; photograph: John Anderson). (d) An ice-dammed lake adjacent to the Russell Glacier (see figure 1) with dry, shallow ponds in the foreground; note the fossil shoreline associated with a previous high stand of this lake (photograph: Ladislav Hamerlik). There are two large herbivores found in the Kangerlussuaq area of SW Greenland, represented by (e) an adult male caribou (photograph: Eric Post) and (f) two adult male musk ox (photograph: Eric Post).

**Figure 3.**
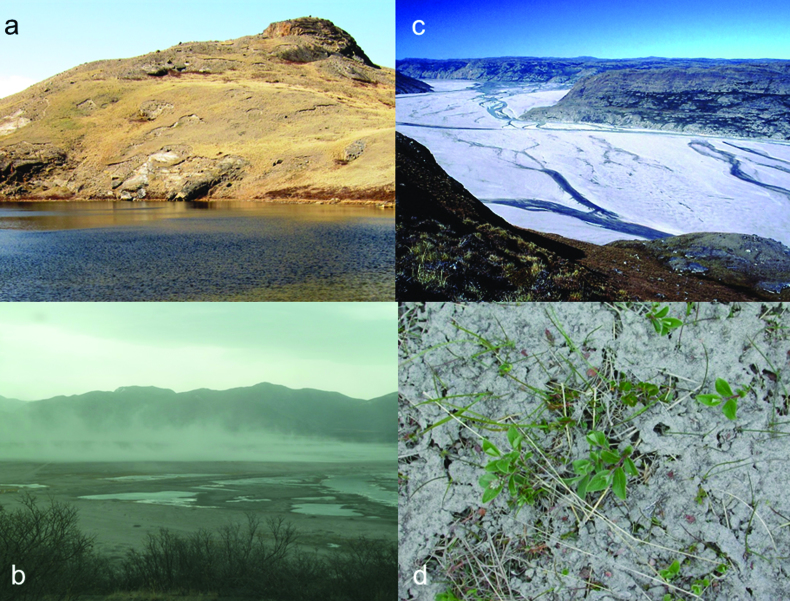
Aspects of aeolian activity around Kangerlussuaq. (a) Wind scouring of the steppe landscape adjacent to the GrIS margin (photograph: John Anderson). (b) A late winter dust storm blowing along Sandflugtdalen (photograph: John Anderson). (c) The sandur immediately below the terminus of the Isunguata Sermia glacier (see figure 1; photograph: John Anderson). (d) Aeolian silt deposited in a lake watershed at an altitude of more than 500 m some 6 kilometers south of the Ørkendalen sandur; the dust was originally deposited on snow in late winter (photograph: John Anderson).

The area along Kangerlussuaq (Søndre Strømfjord in Danish) encapsulates the Arctic in an approximately 150-kilometer, approximately 6000-square-kilometer corridor from the ice sheet itself to the valley and cirque glaciers at the coast. This land mass spanning from the ice-sheet margin to the coast of the Labrador Sea includes a range of landscapes and ecosystems (Figures 2 and 3): glacial and supraglacial; freshly exposed moraines; large outwash plains (sandurs); terrestrial ecosystems that include dwarf shrub tundra, steppe, and snow-bed communities; lakes and ponds that range from organic rich and shallow to dilute and deep and oligosaline and meromictic. Streams range from turbid silt laden rivers draining the ice sheet to oligotrophic and fast flowing in the coastal mountains (see Box 1).

Box 1. **Terms and definitions.**

**Aeolian:** relating to or arising from the action of wind

**Cryoconite:** dust made of small rock particles, soot, and microbes that is found on the surface of a glacier, especially on the bottom of small depressions; causes darkening of ice surfaces

**Dirty ice:** ice discolored by the deposition of atmospheric dust and/or previously entrained silt rising to the surface; important because it alters the surface albedo

**Fluvial:** of or found in a river

**Jökulhlaup:** a type of glacial outburst flood

**Loess:** previously deposited and biologically transformed dust

**Moulin:** a nearly vertical shaft in a glacier, formed by surface water percolating through a crack in the ice

**Paraglacial:** referring to surface processes and landscapes directly conditioned by former glaciation and deglaciation

**Periglacial:** the zone peripheral to glaciers

**Sandur:** outwash plain formed by meltwater from glaciers

**Supraglacial:** anything pertaining to the surface of a glacier but often refers to lakes

**Talik:** region of unfrozen soil or bedrock beneath a lake

The Kangerlussuaq area is located in the continuous permafrost zone, but permafrost conditions vary considerably in the region. Recent borehole measurements and active layer probing show that active layer thickness varies from approximately 30 centimeters in an ice-wedge polygon affected peat bog at 430 meters (m) above sea level near the GrIS margin (Ingeman-Nielsen et al., 2012), to at least 1.8 m in a glaciomarine silty clay deposit at Kangerlussuaq airport (Christiansen et al., 2010). As well as strong local and regional spatial climate and vegetation gradients along Kangerlususuaq, there are also pollutant gradients that reflect rainfall differences as well as ice marginal dynamics. For example, pollution mercury inventories are nearly threefold higher in lakes close to the ice-sheet margin compared with the coast (Bindler et al., 2001).

**Figure 4.**
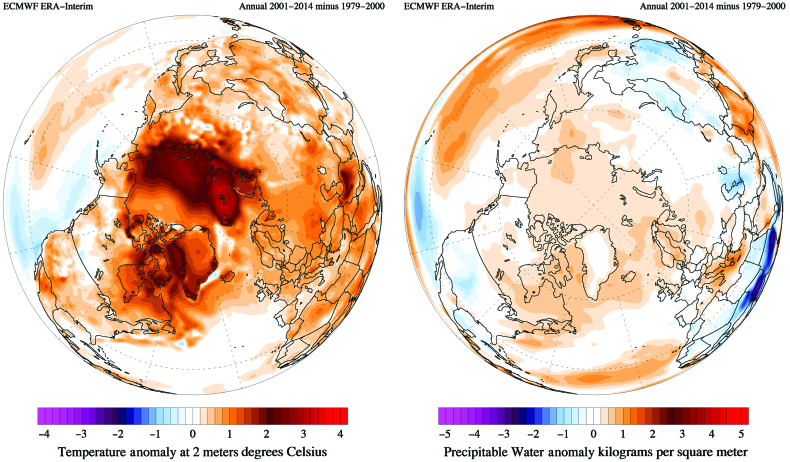
Polar views of recent (a) temperature and (b) precipitation anomalies for the period 2001–2014 compared with 1979–2000, showing the recent climate change in west Greenland, particularly the marked warming. Maps generated via www.ClimateReanalyzer.org (Climate Change Institute, University of Maine).

At Kangerlussuaq airport, the mean annual air temperature (MAAT) was –5.0 degrees Celsius (°C) and –3.9°C for the periods 1974–2012 and 2001–2012, respectively (Mernild et al., 2014). This recent increase in local MAAT (figure 4) is consistent with the MAAT increase observed in Greenlandic coastal synoptic meteorological stations (Hanna et al., 2012), the increasing frequency of warm air temperature extremes (Mernild et al., 2014), and the recent occurrence of extreme melt events in 2010 and 2012 (Tedesco et al., 2011; Hanna et al., 2014). The mean annual precipitation (MAP) at Kangerlussuaq airport was 242 millimeters (1981–2012), showing an insignificant increase in MAP to 258 millimeters in recent years (2001–2012; Mernild et al., 2015) although there has been a change in seasonality. Model simulations based on downscaling of the regional climate model HIRHAM4 coupled with the general circulation model ECHAM5 indicate that from 1950 to 2080 the MAAT and MAP will increase by 3.4°C and 95 millimeters, respectively (Mernild et al., 2011).

## Recent changes within regional landscape geomorphic and ecological systems

### Glacier

The ice-sheet margin at Kangerlussuaq has recently experienced a period of thickening (1980–2000), followed by rapid ice-marginal thinning and recession of outlet glaciers (Knight et al., 2007). Two of the most prominent glacier changes have been the evolution of supraglacial lakes and the reoccurrence of drainage outbursts from a large ice-dammed lake. The number of supraglacial lakes along this part of the western GrIS margin has increased, and almost 30% of the lakes appear to drain in a few days (Fitzpatrick et al., 2014). The supraglacial lakes drain into moulins, which then discharge meltwater to the terminus of the ice sheet, which can contribute to ice marginal lakes. An approximately 1-square-kilometer ice-dammed lake on the northern flank of Russell Glacier (figure 2d) is known to have drained repeatedly from the late 1940s until 1987. After 20 years of lake level stability due to increased thickness of the ice-sheet margin, a jökulhlaup (rapid drainage of ice-dammed lakes) in 2007 marked a renewed cycle of flooding (e.g., Russell et al., 2011). Jökulhlaup events have resulted in downstream floods almost every year since 2007 during the late summer. Also, intense ice-sheet melt in July 2012 caused the Watson River to rapidly reach the highest level ever recorded since records began in the 1940s. Both types of glacier floods affect downstream proglacial geomorphology, sedimentary systems and biological communities at Kangerlussuaq (e.g., Carrivick et al., 2013). In brief, these geomorphological impacts include delta formation into lakes, channel bank erosion, channel bedrock incision, and sediment deposition in terrestrial and lacustrine basins and into the fjord.

### Terrestrial

Since observational monitoring of plant phenology in Kangerlussuaq began in 1993, the start of the plant growing season has advanced by approximately 20 days (Kerby and Post 2013, Post 2013). Phenological advancement has varied considerably among plant species, with graminoids and early-emergent forbs displaying the greatest advancement, and deciduous shrubs the least pronounced advancement, in the timing of spring green-up. Despite slower rates of phenological advance, canopy cover of the two dominant species of deciduous shrubs, *Betula nana* and *Salix glauca*, has increased near Kangerlussuaq, ostensibly in relation to local warming (Post et al., 2013). Plot-scale CO_2_ flux measurements indicate that such increases have the potential to substantially increase net ecosystem carbon uptake (Cahoon et al., 2012). Increases in deciduous shrub cover in the area may, however, be checked on occasion by outbreaks of the caterpillar larvae of a noctuid moth, *Eurois occulta*, of which there have been two since 2002 (Avery and Post 2013). Such outbreaks may increase in severity in the Kangerlussuaq region and other parts of the Arctic with future warming.

Animal populations have also changed in recent decades. Daily censuses of the two resident species of large herbivores inhabiting the area, caribou (*Rangifer tarandus*) and muskoxen (*Ovibos moschatus*), have been conducted annually throughout the reproductive seasons of both species at a long-term study site 25 kilometers east of Kangerlussuaq (Post 2013). These counts indicate that the annual maximum number of caribou observed at that site have declined from a peak of nearly 600 in 2006 to 119 in 2015, whereas the annual maximum number of muskoxen has fluctuated between approximately 15 and 50 and may be increasing slowly. The endemic Greenland white-fronted goose, *Anser albifrons flavirostris*, has been declining since peaking at 35,600 in 1999 (Stroud et al., 2012), whereas the Canada goose, *Branta canadensis interior*, has probably been breeding in West Greenland since at least 1863 but has increased in range and abundance in recent years, reaching over 500 in recent years (Fox and Glahder 2010).

### Aquatic

Limnological surveys were initiated in 1996 (Anderson NJ et al., 2001), prior to the recorded onset of regional warming, and since then, there have been a number of systematic changes. Like the majority of Arctic lakes, those around Kangerlussuaq are nutrient poor (total phosphorus, P, is less than 7 micrograms per liter, μg per L; total N ranges 300–800 μg per L; Whiteford et al., 2016) but are major C stores at the landscape scale (Anderson NJ et al., 2009). Nitrate is often low, with the exception of the coastal lakes, where it is notably higher in the spring from a strong N-pulse derived from melting snow pack. Stable isotope analyses of this NO_3_ suggest it is enhanced by atmospherically derived reactive N deposition. The spatial gradient in nutrients and their seasonal availability results in pronounced patterns in nutrient limitation (Whiteford et al., 2016).

Another notable change is the increasing lake levels in a number of the oligosaline lakes at the head of the fjord, where lake levels have increased by up to 2.5 m over the last 12 years, as has been evidenced by drowned shrub tundra along the lake shores. Many of the inland lakes have high dissolved organic carbon (DOC) concentrations (approximately 40–100 milligrams per L) because of the long retention times and evapoconcentration over centuries (Anderson NJ and Stedmon 2007). Regional declines have been observed in DOC (by 60%) in a number of freshwater lakes in the period 2000–2014 (Saros et al., 2015); there have also been increases in sulfate but not conductivity or chloride. This decline in the DOC pool coupled with recent decreases in C burial rates in this area suggests major changes in C cycling over recent years. Moreover, the change in DOC concentration coupled with rising air temperatures will change thermal stratification patterns with associated implications for primary production (Saros et al., 2015).

## Linkages across a paraglacial landscape

**Figure 5.**
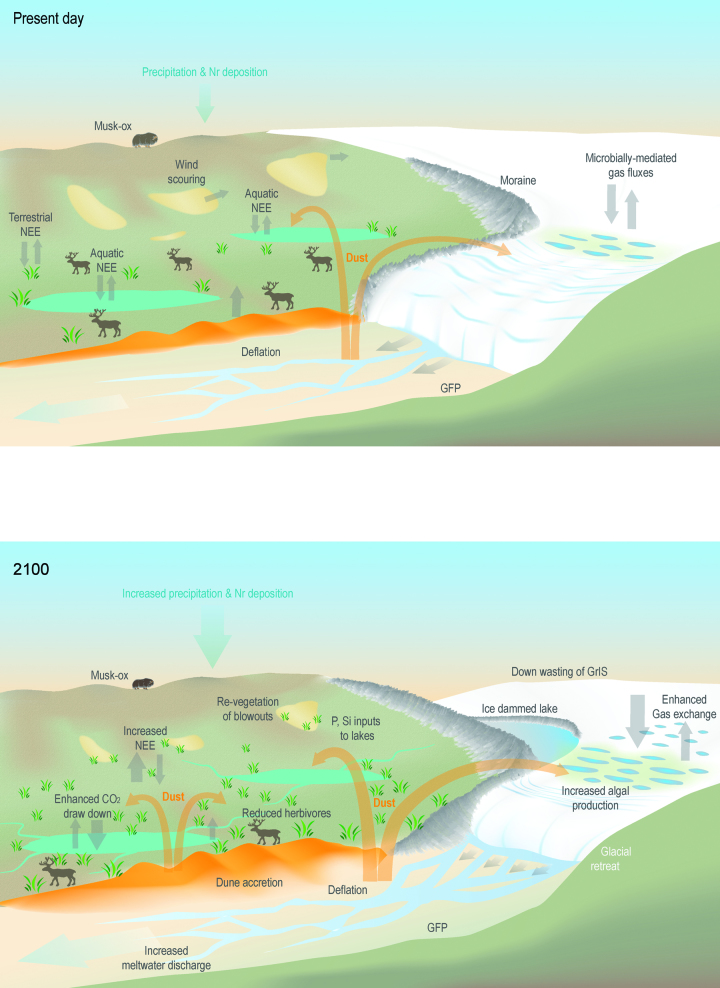
A schematic representation of the possible changes in key landscape features and geomorphic and ecosystem processes in the paraglacial of the Kangerlussuaq area between the present day (upper panel) and 2100 AD (lower panel). Key geomorphic or landscape features include the sandurs (glacio-fluvial outwash plains [GFP]), dunes, blowouts, moraines, and ice-marginal lakes. Note the substantial retreat of the outlet glacier, the development of ice-dammed lakes, increased biological activity on the glacier or GrIS (with associated greenhouse gas [GHG] fluxes) and hydrological loss; and dune expansion and the transfer of aeolian material from the sandurs to adjacent terrestrial and limnic ecosystems. On land, deepening active layer depth may increase surface-water accumulation, with GHG fluxes, the expansion of shrub tundra, and the revegetation of blowouts. Lakes will be ice free for longer with altered gas exchange.

The complex interactions between the GrIS and adjacent ecosystems are not immediately apparent given their well-delineated boundaries (i.e., ice, water, and tundra) and despite their proximity: It is possible to transition from Arctic steppe or tundra to glacial ice in meters (figure 2c). Moreover, although there is considerable hydrological discharge from the GrIS annually there is a hydrological disconnect between the ice sheet and adjacent terrestrial ecosystems (figure 5). Similarly, the low annual precipitation and the presence of continuous permafrost suggest that hydrological linkages between tundra soils and aquatic ecosystems are presently limited. Synthesizing research in the Kangerlussuaq area over the last two decades, however, highlights the complex interactions between glaciology, the terrestrial geomorphic system and terrestrial ecosystems (both tundra and limnic). These interactions operate at a range of ecological or organismal (microbes to reindeer) and spatial (e.g., the migration of the Greenland white-fronted goose from Greenland to the British Isles and of Canada geese to North America) and temporal scales (seasonal C fixation by microbes on the ice sheet to long-term C sequestration by lakes). Here, we highlight some of the major interactions.

Seasonal melt events and jökulhlaups that cause widespread flooding deposit fine (less than 2000 μm) sediments across the sandur. Following recession of the water, these deposits desiccate rapidly. Strong winds can entrain the sediments causing dust storms and forming localized dunefields, effectively linking the glaciofluvial and aeolian systems (figures 3 and 5). Bullard and Austin (2011) described rapid deflation of jökulhlaup deposits leading to intense dust storms in the valley. There are no year round measurements of modern aeolian flux or deposition rates for the region, but process studies of dust flux on the sandur suggest summer transport rates of up to 0.082 grams per meter width per second (g per m w per s; Bullard and Austin 2011).

Dust storms can reach several hundred meters above the sandur plain (figure 3d). Dust deposited on the surface of the ice-sheet ablation zone preferentially absorbs solar radiation, and melts down into the ice surface. These debris accumulations may be concentrated (in cryoconite holes) or dispersed (as “dirty ice” or ice algae; Yallop et al., 2012), but are biological “hot spots” on an otherwise inhospitable glacier surface. Recent surveys highlight the sheer scale of active photosynthetic microbial communities in cryoconite holes (Yallop et al., 2012). Meltwater flushes through the ice algae and debris, initially in small, interlinked water veins and eventually in supraglacial streams that lead to supraglacial lakes or moulins.

Aeolian material is also deposited on soils and directly into lakes across the region. Modern dust deposition rates in lake catchments above the floodplain are around 70 g per square meter per year (Willemse et al., 2003). Evidence of the persistence of this process in the past can be found in lake (Anderson NJ et al., 2012) and peat (Willemse et al., 2003) records throughout the Kangerlussuaq region and in the widespread loess deposits in interior western Greenland. These paleo-records suggest that aeolian activity has varied during the Holocene and that the magnitude and frequency of aeolian processes is closely linked to both ice-sheet hydrology and proglacial geomorphology, which control sediment supply and availability. The strong katabatic winds also cause local erosion on exposed slopes (figures 3a) and these deflation patches are widespread closer to the ice sheet, driven by strong winds (more than 20 m per S) blowing off the ice sheet. This erosion represents local re-working and translocation of soil and loess, which has implications for local C and nutrient budgets.

## How are these linkages across the landscape changing?

### Water

Although the vast majority of ice-sheet melt is routed to the ocean, a substantial portion is routed first across the terrestrial landscape, providing subsidies of water and sediment. For Greenland as a whole, 69% of the runoff to the surrounding seas originated from the GrIS and 31% came from outside the GrIS (from rain and melting glaciers and ice caps; Mernild and Liston 2012). For the GrIS specifically as a whole, about 75% of this proglacial meltwater flows into rivers, and the remainder enters lakes at the margins of the ice (Lewis and Smith 2009). These ice-marginal lakes may increase in number and/or size with increased melt of the GrIS associated with a warming climate (figure 5). Proglacial lakes and rivers fed by land-terminating glacial lobes receive large plumes of sediment-laden meltwater. Sediment deposited by these rivers and lakes is the primary source of the fine sediment transported in aeolian processes. An increase in the frequency of jökulhlaups, as was predicted by Russell and colleagues (2011), may consequently lead to an increase in sediment deposition (figure 5) and therefore an increase in the magnitude and frequency of dust storms, assuming sufficient aeolian transport capacity.

In periglacial areas removed from the direct influence of the ice sheet, the routing of water across the landscape is affected by seasonal and interannual changes in permafrost dynamics, as well as by snow accumulation and melt, which are strongly influenced by sublimation (Johansson et al., 2015). Global climate simulations suggest a widespread increase in permafrost thaw in the twenty-first century, with increased movement of water through the subsurface and, in some regions, increases in precipitation that outpace evaporation increase (Lawrence and Slater 2005). These changes in water movement through the active layer will affect weathering rates and the transport of solutes, including major cations, metals, and trace elements (Jessen et al., 2014), and inorganic and organic carbon (Schuur et al., 2015). Therefore, changes in permafrost cover and duration are likely to influence the hydrology and biogeochemical dynamics of both terrestrial and aquatic ecosystems (figure 5). For example, in Arctic Alaska, deepening of the active layer and melting of nutrient-rich permafrost has enhanced the growth of herbaceous plants, which have encroached on shallow ponds and reduced open water cover by approximately 17% since 1948 (Andresen and Lougheed 2015). This unexpected dynamic further emphasizes the difficulty of predicting future hydrologic mass balance on a landscape scale.

Measurements and coupled hydrologic mass balance modeling in the Kangerlussuaq area suggest that catchment water balance is spatially quite variable, in part as a result of the steep precipitation gradient from the ice sheet westward toward the oceanic moisture source (Johansson et al., 2015; Mernild et al., 2015). This variability has important implications in terms of generalizing about terrestrial and aquatic ecosystem responses to climate change. Water budgets of catchments and lakes isolated from direct impact of the GrIS will be dependent on the balance between local precipitation and evaporation, including changes in sublimation during winter and spring. In the Kangerlussuaq area, some lakes also exchange water with a deep groundwater system through taliks, although the amount is very small (less than 5%; Johansson et al., 2015), which may modulate the direct impacts of climate change. Work on an East Greenland glacier noted that groundwater was geochemically enriched compared with glacial meltwater and was clearly distinguishable using stable isotopes (δ^18^O), suggesting that groundwater inputs from a retreating GrIS can be traced (Kristiansen et al., 2013). Weathering rates will increase with warming and alter groundwater geochemistry (Anderson SP 2007) changing the major ion and nutrient inputs to newly formed lakes (cf increased alkalinity input to Toolik Lake (Hobbie JE and Kling 2014). The groundwater influence will be increasingly mediated by soil development (cf. Glacier Bay; Engstrom et al., 2000). Work in West Greenland is limited, but an analysis of the streams around Kangerlussuaq revealed diverse geochemistry (compared with streams on Disko Island) with relatively enhanced phosphate concentrations (Friberg et al., 2001), suggesting that lakes may be positively affected by groundwater-influenced hydrological inputs as the GrIS retreats.

In the past, changes in regional temperature and precipitation have produced considerable lake level changes. Geomorphic and paleolimnological records in the Kangerlussuaq region document lake-level rises of 1.3 m above modern during pluvial periods of the last 2000 years and up to 18 m of lake level decline during the mid-Holocene, when independent data suggested regional summer warming of 2°C–3°C (McGowan S et al., 2003; Aebly and Fritz 2009). Changes in climate seasonality are likely to have prominent impacts on hydrologic mass balance.

### Carbon

**Figure 6.**
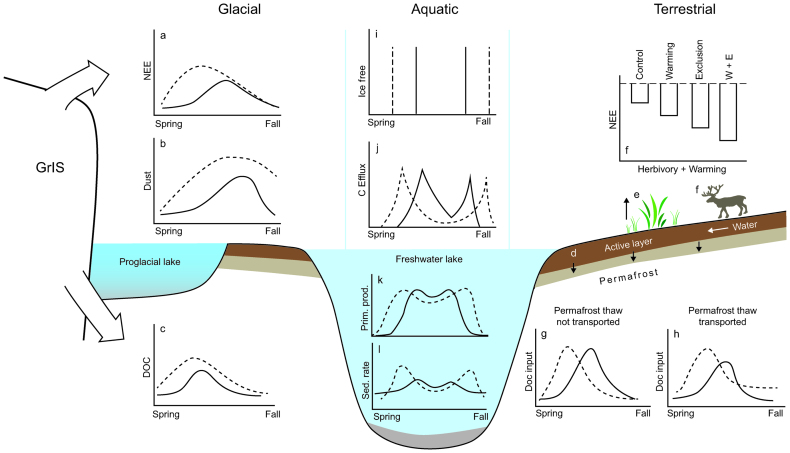
A conceptual diagram showing the C-cycle processes and interconnectedness among glacial, terrestrial, and aquatic ecosystems in the Kangerlussuaq region today and in the future. Under a warming climate (dashed lines), supraglacial net ecosystem exchange (NEE) is expected to reach peak flux earlier in the growing season (a), driven by earlier and greater input from alluvial deposits as the glaciers retreat (b). This may result in greater DOC export to proglacial lakes through the active layer (c). Warming may lead to greater lake terrestrial NEE, depending on the depth of permafrost thaw (d), shrub expansion dynamics (e), and the presence of herbivores (f; plot adapted from Cahoon et al., 2012). Changes in permafrost and terrestrial NEE will likely affect the timing and magnitude of DOC input to lakes, depending on whether permafrost meltwater acts as a vector and is transported to lakes (g, h). Similar to pan-Arctic observations, we expect warming to extend the ice-free period of lakes (i) and lead to earlier peak CO_2_ flux in spring and later CO_2_ flux in fall (j). A similar pattern in primary productivity (k) and sedimentation rates (l) is likely to occur under warming in response to an extended growing season.

Changes in climate have begun to alter the carbon (C) cycle linkages among glacial, proglacial, aquatic and terrestrial systems throughout the Kangerlussuaq region in several ways (figure 6). Increased spring warming and a longer melt season has expanded the melt zone across the GrIS (Tedesco et al., 2012), increasing the region available for autotrophic microbial communities in cryoconite and bare ice to colonize and grow. An earlier start to the spring melt season will alter patterns of microbial cycling in supraglacial environments, and potentially increase rates of net ecosystem production (NEP) and DOC export from the glacier (figure 6a; Yallop et al., 2012; Lawson et al., 2014). Increased supraglacial ablation rates will also increase the supply of sediment melting out from glacial ice (Stibal et al., 2008) because there is strong evidence that much of the DOC exported from glaciers is derived from microbial production in the supraglacial environment (Bhatia et al., 2013; Lawson et al., 2014). Increased microbial production associated with spring warming will therefore result in greater DOC fluxes and earlier export of DOC and particulate organic carbon (POC) to the subglacial environment and glacial outlet rivers (figure 6b; Lawson et al., 2014), as long as increased glacial melt does not flush supraglacial microbes from the GrIS and offset DOC production (Stibal et al., 2012). However, changes in DOC and POC export from the sub- and supraglacial environments are unlikely to directly influence nearby lakes because of the severe disconnect between glacial outwash and terrestrial systems. In fact, organic C produced in supraglacial areas has little effect on aquatic and terrestrial ecosystems in the Kangerlussuaq area until sediments are deposited on riverbanks where it is subsequently carried across the landscape by aeolian transport (figure 6c). Estimates of organic C fluxes from aeolian deposition (approximately 50 milligrams C per m per year) are about three orders of magnitude lower than NEP in nearby proglacial lakes and are therefore unlikely to significantly affect NEP in these ecosystems. These aeolian carbon fluxes are, however, similar to rates of NEP on the surface of glaciers in the area (5 to 149 milligrams C per square meter per year; Stibal et al., 2012).

In a warming permafrost landscape, the composition of soil and lake DOC pools are likely to change in response to shifts in terrestrially derived C and internal lake dynamics. DOC is a complex mix of compounds sourced from degraded terrestrial and aquatic primary productivity and serves as a cross-ecosystem material that is dependent on direct and indirect climatic influences. Decreasing DOC concentrations in the lakes over the past decade (Saros et al., 2015) may result from multiple drivers including decreases in terrestrial subsidies and/or increased sulfate deposition. Lake-water sulfate concentrations have increased across the Kangerlussuaq region over the past decade (Saros et al., 2015); it is known that sulfate deposition can lower the pH of soils and increase retention of DOC in the watershed. Ultimately, the linkage between aquatic and terrestrial systems will depend largely on landscape hydrological connectivity. Currently, there is a strong link between aquatic and terrestrial DOC early in the growing season when spring snowmelt runs off into lakes. However, this relationship weakens over the growing season as soils dry and recharge from precipitation is low. Highly colored, diluted, and possibly photooxidized (Osburn et al., 2001) DOC was recovered from lakeshore wells in the Kangerlussuaq region in early June; however, the same wells were dry and DOC content very low in early August. Under scenarios of increased spring warming, we expect an advancement in the timing and possibly the magnitude of this pulse of terrestrial DOC, depending on how climate change affects surface and subsurface hydrology. In one scenario, if rising temperatures lead to an increase in permafrost degradation throughout the growing season, more DOC will be transported into surrounding lakes (figure 6d). Alternatively, greater evapotranspiration may decouple permafrost meltwater from surrounding lakes, resulting in a loss of watershed connectivity and limit total DOC transport (figure 6e).

Shifts between allochthonous and autochthonous C sources in lakes will alter the composition of the DOC pool, which may, in turn, alter competitive interactions among microbial species (Crump et al., 2003) and restructure community assemblages. In a highly seasonal environment such as southwest Greenland (and the Arctic), changes to the temporal inputs of soil DOC will likely lead to seasonal shifts in lake microbial communities toward fast growing microorganisms that are able to rapidly use DOC inputs from soils. This process may be amplified as ice-free durations of Arctic lakes have increased over the last century (figure 6f) in response to climate warming (Magnusson et al., 2000). Extended ice-free seasons have reduced underice mineralization and net C emissions in some regions (figures 6g and 6h; Finlay et al., 2015) and increased C burial in lake sediments (figure 6i).

Long-term changes in plant community composition driven by geomorphic processes (Heindel et al., 2015), climate warming and/or large herbivores will also influence the link between terrestrial and aquatic environments by altering terrestrial C pools. The low-shrub tundra in the region has displayed divergent responses to warming when large herbivores were excluded. For instance, warming without large herbivores reduced species diversity of the plant community, whereas herbivory maintained this diversity even under warming (Post 2013). Moreover, when warmed and excluded from herbivory by caribou and muskoxen, the tundra acted as a C sink (200 g C per square m), but the area accumulated less than half that amount when exposed to herbivores (Cahoon et al., 2012). However, when exposed to herbivores, graminoids continued to dominate and the communities accumulated less than half the amount of C relative to exclosed sites (Cahoon et al., 2012), providing evidence of a strong trophic interaction that can limit terrestrial C uptake (figure 6j). With greater leaf area, shrubs tend to fix more C than herbaceous species do (Cahoon et al., 2012), whereas shading from a closed canopy can keep soils cool and limit respiratory losses. Although shrub tundra acts as a stronger C sink, woody stems provide a more recalcitrant source of C than herbaceous communities (Hobbie SE 1996); therefore, greater C uptake associated with shrub expansion may not necessarily lead to greater DOC available for transport to aquatic environments, at least in the short term. Nevertheless, changes in the relative abundance of deciduous shrubs will influence the quality and quantity terrestrial C pools that serve as an upstream source of DOC for aquatic environments.

Changes in plant community composition in response to warming will directly alter the terrestrial C cycle, but also affect the fraction of belowground DOC available for transport. Whether the link between terrestrial and aquatic environments will strengthen or weaken in response to warming will depend on the synergy of watershed connectivity (associated with changing precipitation and thawing permafrost) and the relative fraction of labile DOC (Frey and McClelland 2009). Changes in terrestrial DOC production and meltwater delivery of terrestrial C (either from permafrost or snowmelt) are crucial interacting factors that warrant further investigation in southwest Greenland and throughout the Arctic.

### Nutrients and other elements

**Figure 7.**
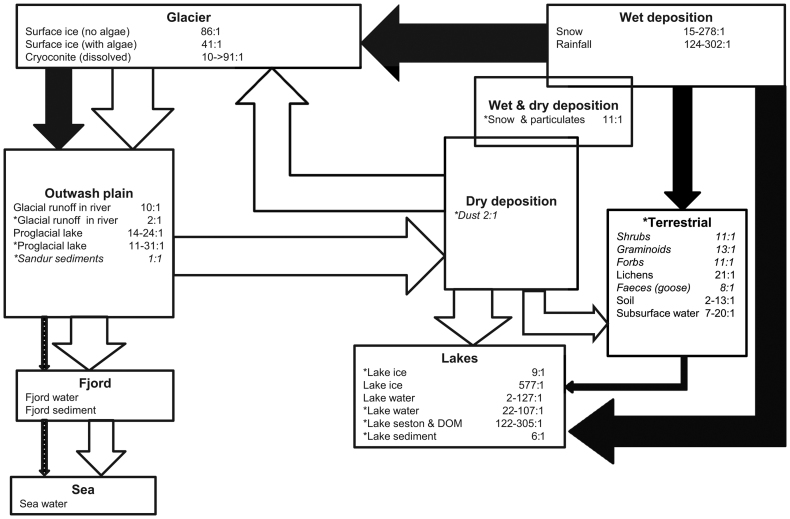
A conceptual diagram of the atomic N:P ratios and cross-system transfers of nitrogen (black arrows) and phosphorus (white arrows) among components of the Kangerlussuaq landscape. The thickness of the arrows indicates the degree of influence that the transfer has on N:P ratios of the receiving system (no or negligible influence = no arrow, and categories indicate small, medium, and large influence). N:P ratios are measured on the dissolved inorganic (generally bioavailable) fraction, unless indicated with an asterisk, where total digested nutrient concentrations were measured. Values in standard text are data derived directly from Kangerlussuaq, whereas those in italics derive from published arctic or subarctic values. Data sources are surface ice with and without algae; cryoconite waters (N data from Stibal et al., 2012); glacial runoff waters (Lawson et al., 2014, Hawkings et al., 2016); proglacial lake waters, sandur sediments, and dust (Wientjes and Oerlemans 2010); wet deposition and snow with particulates; shrubs, graminoids and forbs measured in subarctic Sweden (Gusewell 2004); lichens; goose feces from Svalbard (van der Wal and Loonen 1998); subsurface water; and lake data (Whiteford et al., 2016).

The hydrological disconnect between the glacial outwash plains and much of the terrestrial landscape is a key constraint on nutrient cycling within the Kangerlussuaq region (figure 7) although this may change as the GrIS retreats, permafrost melts and talik zones deepen. This separation creates a prominent role for aeolian and atmospheric transport pathways between the ice sheet and adjacent land surface with important consequences for nutrient stoichiometry. N is typically transported in dissolved forms, whereas P adsorbs to sediments and particles, and so waterborne P transport is generally efficient within energetic sediment-laden riverine environments (e.g., the fluvial outwash plain) but more limited where stream flows are slow or dominated by seepage (e.g., on the terrestrial land surfaces around Kangerlussuaq, where the low precipitation-to-evaporation ratio limits the development of streams and rivers). On the ice-sheet surface, microbial nutrient processing hotspots include “dirty ice” (Yallop et al., 2012) and cryoconite holes (Stibal et al., 2012, Telling et al., 2012). Clean ice has inorganic N:P ratios of 86:1, indicating that N is sourced via wet deposition (Hastings et al., 2009). Therefore, dust (estimated N:P ratio of 2:1) is probably important as a P fertilizer to facilitate microbial growth (Mindl et al., 2007). Where algae grow directly on the ice surface, inorganic N:P ratios are lower (41:1), probably indicating patches where P fertilization through dust accumulation has modified the local environment (Stibal et al., 2010) and N has been sequestered by algae (Yallop et al., 2012). The highly variable N:P ratios in cryoconite holes (10–91:1), where N_2_-fixing cyanobacteria are common, indicate strong and divergent biological modification of inorganic nutrients within these environments (Telling et al., 2012). Areas of dust accumulation may therefore stimulate localized “patchy” development of algae on ice with significant increases in pigmented biomass (Yallop et al., 2012; Lutz et al., 2014).

Glacial runoff waters have much lower N:P ratios (dissolved 10:1; total 2:1) than on the ice surface, suggesting biogeochemical modification on the passage through the subglacial system (Yde et al., 2010), where microbial processing and the addition of glacial flour increase the bioavailable P (Stibal et al., 2012; Wadham et al., 2013). Discharge into proglacial lakes enhances N:P ratios (to 14–24:1) because they often have abundant cyanobacterial N_2_ fixing communities and probably remove P from the water through rapid sedimentation of organically and inorganically bound particulates (McGowan HA et al., 2008). In contrast, the sediment-laden river waters (2–15 kilograms, kg, per cubic meter (Cowton et al., 2012) efficiently transport particulate-bound P and dissolved N to the sandurs, fjord and coastal regions (Hawkings et al., 2015). Nutrient ratios within the river–fjord system are most likely modified primarily through physical and chemical processes because the turbulent conditions are inhospitable for the development of many biota. Apart from some N uptake and sedimentation by primary producers within the fjord ecosystem (NO_3_-N concentrations are 116 μg per L; Hawes et al., 2012), N is probably exported in dissolved form to the coastal regions (Hawkings et al., 2015), with implications for sustaining productivity of plankton and ice algae in coastal waters.

Inorganic N:P ratios within snowfall (15–278:1) indicate significant N delivery as wet deposition (0.8 kg N per hectare per year; Christopher Curtis, School of Geography, Archaeology and Environmental Studies, University of Witwatersrand, Johannesburg, personal communication, 19 February 2015), whereas snow with particulates has a lower ratio (11:1), indicating enrichment with P, most likely from dust. Direct atmospheric deposition of reactive N to terrestrial and aquatic ecosystems has increased over the last 50–100 years (Hastings et al., 2009), whereas N deposited on the GrIS is processed extensively prior to its transfer to adjacent ecosystems (see above). Therefore, direct wet and dry deposition are major determinants of terrestrial nutrient availability, and nutrients are most mobile during spring snowmelt when ephemeral rivers activate (Johansson et al., 2015). Soil N:P ratios of 2–13:1 around Kangerlussuaq (c.f. mean global ratios 13:1) suggest relative P enrichment, consistent with observations that soil surface layers at Kangerlussuaq are influenced by aeolian silt deposition (Nielsen 2010) and that P availability is generally greater in geologically young soils. The variable soil N:P ratios in Kangerlussuaq also suggest heterogeneity of nutrient delivery and processing, as has been demonstrated by the mosaic of vegetation types that are also strongly constrained by water availability (Thing 1984). Herbivore grazing can modify nutrient cycling locally, such as reindeer use urine and feces to fertilize local areas and encourage *Poa pratensis* growth (Thing 1984). Because of snowmelt percolation, subsurface soil waters are enriched in N during the spring, mostly as organically bound forms (N:P ratio of 25:1), but the ratio reaches 7:1 by late summer when N availability declines.

Lake measurements show a spring snowmelt N pulse, delivered predominantly as ammonium but with some nitrate (Whiteford et al., 2016). Lake bioavailable N becomes depleted throughout the growth season. Importantly, the extremely high N:P ratios within lake seston and organically bound fraction (122–305:1) indicate both P removal through sequestration into lake sediments (N:P ratio 6:1) and the retention and accumulation of N in lakes, most probably incorporated into recalcitrant dissolved organic material that accumulates slowly in the closed lake basins (Anderson NJ and Stedmon 2007). This long-term N retention in lake waters probably explains why a period of pelagic P limitation occurs in the spring under ice (Whiteford et al., 2016) when algal growth begins, but lakes are sealed from atmospheric P (dust) sources. Because of limited surface outflow from these lakes, the primary nutrient transfer pathways between lakes and the terrestrial areas are probably subsurface flows (Johansson et al., 2015) and transfer by biota. For example, chironomids can fertilize soils adjacent to lakes when they emerge as adults (an estimated potential local load of 0.5 kg N per hectare per year N and 0.05 kg P per hectare per year N), and water birds such as geese and ducks may be significant vectors (van der Wal et al., 2007). Canada Geese commonly nest and forage along lake shores considerably more than white-fronted geese, with consequences for nutrient cycling and aboveground primary production as well as species composition of vegetation. Later, during molt, both species of geese specialize on repeated grazing of grasslands and open moss mats within 40 m of the water's edge, habitually returning to the water to rest and preen in safety from terrestrial predators. Fecal deposition of material obtained from foraging in terrestrial habitats in the vicinity of lakes is therefore deposited in or near the water in the form of organic material and the products of protein metabolism, which includes soluble N compounds. Therefore, the general increase in molting goose numbers (regardless of species composition) in the vicinity of lakes in this area over the last three decades in summer has likely had a major effect on vegetation communities and their carbon and N dynamics. Because the density of lakes is high in this region (more than 20,000 lakes) and there is a significant discrepancy between N:P ratios in lake and terrestrial organic matter, there is great potential for trophic interactions between lake and land to modify nutrient ratios.

### Broader anthropogenic influences

Interactions between climate, dust production, atmospheric pollutants and trophic dynamics have the potential to significantly modify a number of regional biogeochemical linkages in the future. Increasing GrIS surface melt and subsequent Watson River runoff in the future (Mernild et al., 2011) will increase nutrient export from the inland ice to the fjord and coast and expand deposition of dust within the sandur (Yde et al., 2014). Future N deposition will depend on global economic development and legislation, but is likely to increase, with implications for all elements of the Kangerlussuaq system. Together, these changes will probably alter the N:P delivery to terrestrial and ice-sheet areas. Plant phenological advance has influenced trophic interactions between producers and consumers, possibly contributing to a decline in calf production in the local caribou population (Kerby and Post 2013). The effect of hunting pressures also needs to be considered. Heavy metal contaminants such as lead and mercury have been recorded in western Greenland since AD 1800 and AD 1900, respectively (Bindler et al., 2001; Lindeberg et al., 2006). A particular consideration is the special meteorological conditions in Kangerlussuaq that appear to have concentrated mercury (three- to fivefold increases in concentrations and accumulation rates over the last century) and the effects on biota in this area (as opposed to the marine realm) are not extensively studied to date. Use of ^206^Pb or ^207^Pb isotope ratios in lake sediment led Bindler and colleagues (2001) to conclude that Eurasia was the dominant source of atmospheric pollutants (including Hg and Pb) in SW Greenland. Little is known of how delivery of other micronutrient elements (e.g., zinc, copper, manganese, magnesium, cobalt, and boron) may change in the future, with implications for microbial growth on the ice sheet and lakes. As we noted above, sulfate concentrations have increased in Kangerlussuaq lakes, raising intriguing questions about the potential for a marine–terrestrial–freshwater linkage. Oceanic phytoplankton produce dimethyl sulfate (DMS), which is aerosolized, transformed into methanesulfonic acid (MSA), and transported onto landmasses. Sharma and colleagues (2012) noted significant increases in MSA over the past decade in the Arctic, indicating a possible role for biogenic sulfate delivery and deposition across the study area. Together, these examples indicate how global change processes can initiate localized influence on the biogeochemistry of the Kangerlussuaq region.

### Interregional comparisons

Hange in the Kangerlussuaq area will be strongly influenced by GrIS dynamics, and although this is an analogue for changes as the Fennoscandinavian and Laurentide ice sheets decayed at the end of the last glacial period, future change in much of the Arctic is not similarly affected. All of the Arctic will be affected by extreme climate events (e.g., late winter warming) although there will be spatial and temporal variability. The future of the tundra systems north of the Brooks Range in Alaska has been reviewed by Hobbie JE and Kling (2014), who highlighted enhanced soil microbial activity altering nutrient budgets and greater thermokarst activity; the latter will not be so marked in the Kangerlussuaq area, where soils are thinner. In comparison with much of the Arctic, the lower precipitation levels may restrict greening in Kangerlussuaq, whereas maritime effects may be greater along the north extremes of Alaska, Arctic Canada, and Siberia as sea ice cover continues to decline (Post et al., 2013). We have highlighted the importance of linkages between the geomorphic and ecological systems, which are mediated by the airshed through dust dynamics. Dust deposition is extensive in parts of Alaska (Bullard et al., 2016), but its ecological impact probably requires greater consideration both north of the Brooks Range and across much of Siberia, which are affected by extraregional sources (i.e., dust from northern China).

## Conclusions

The Kangerlussuaq area of southwest Greenland is changing rapidly: Regional warming is driving increased seasonal melt on the ice sheet, altering phenology and changing landscape hydrology with associated effects on lakes and ponds. Some of these changes are interacting in unpredictable ways (meltwater pulses and dust production), whereas others may have cascading effects, such as altered herbivore densities on tundra vegetation and soil C dynamics. Our understanding of the effect of altered herbivore abundance (geese as well as large herbivores) on nutrient budgets and C fluxes is still preliminary as is the reverse subsidy of terrestrial secondary production by aquatic production.

Superimposed on climate-driven processes are chronic, subtle changes in atmospheric pollutants, which may have synergistic effects, such as altered N loading on soil nutrient pools and therefore soil microbiology. This preliminary synthesis has highlighted the need to better constrain atmospheric nutrient input rates (dust, reactive N) to the ice sheet, lakes, and soils, as well as their processing by the glacier and soil subsystems. The effect of the meteorological conditions at the ice-sheet margin on pollutant (and nutrient) deposition requires clarification. The cross-system linkages between geomorphic processes (dust production, glacial erosion) and ecosystems that influence regional biogeochemical cycling may change in unpredictable ways because of the broader regional climate changes that go beyond temperature, such as altered seasonality of precipitation, wind speeds, and evapotranspiration. The effect of future changes in precipitation (e.g., seasonality and snow cover extent) and N loading represent an important but poorly constrained aspect of altered regional biogeochemistry. Changes to the groundwater system (and therefore nutrient and major ion fluxes) and its effect on lakes and streams need to be assessed.

Research in the Kangerlussuaq area is ongoing and tracking changes (e.g., lakewater DOC, altered phenology, and ice-sheet ablation) in real time, but there is clearly a need to link quantitatively the numerous paleoecological records in this area with contemporary measurements. This fusion of time series will provide a more rigorous definition of natural background conditions against which twenty-first-century change can be compared. The effects of these complex linkages for regional carbon dynamics and sequestration highlight the need to take a holistic view of the changing climate, geomorphic, and ecological systems as they influence both aquatic and terrestrial communities in the twenty-first century as the Arctic continues to change.
